# Benchmarking cell-type deconvolution in cross-platform transcriptomic data

**DOI:** 10.1186/s13059-026-04222-8

**Published:** 2026-08-13

**Authors:** Aakanksha Singh, Pinar Cakmak, Jennifer H. Lun, Jadranka Macas, Karl H. Plate, Yvonne Reiss, Jonathan Schupp, Katharina Imkeller

**Affiliations:** 1 Goethe University, Faculty of Medicine, Institute of Neurology (Edinger Institute), Frankfurt, Germany; 2 Frankfurt Cancer Institute (FCI), Frankfurt, Germany; 3 University Cancer Center (UCT), Frankfurt, Germany; 4 Faculty of Biology, Goethe University Frankfurt, Frankfurt, Germany; 5 Goethe University, University Hospital, Dr. Senkenbergische Institue für Pathologie, Neuropathologie und Humangenetik, Frankfurt, Germany; 6 Present Address: Current address: Heidelberg University Hospital, Neuropathology, Institute for Pathology, Heidelberg, Germany; 7 Present Address: Current address: Chemotherapeutisches Forschungsinstitut Georg-Speyer-Haus, Frankfurt, Germany

**Keywords:** Deconvolution, Cross-platform, Experimental factors, Benchmarking, Transcriptomics

## Abstract

**Background:**

Transcriptomic data from diverse measurement technologies are widely used to study tissue heterogeneity. Cell-type deconvolution, which resolves mixed transcriptomic signals into cellular components, is a key analytical approach. However, achieving accurate deconvolution across platforms remains challenging due to platform-specific experimental and technological biases.

**Results:**

We systematically benchmarked deconvolution performance using real-world cross-platform datasets and simulated data modeling distinct technological features. SpatialDecon and cell2location demonstrated the most reliable and consistent performance across both simulated and experimental settings across a broad range of technological biases.

**Conclusions:**

Our results highlight how the different deconvolution tools are affected by data properties that depend on technological differences between transcriptomic platforms. Moreover, we provide practical guidelines for selecting computational methods dependent on experimental design for robust deconvolution of cross-platform transcriptomic data.

**Graphical Abstract:**

**Figure:**
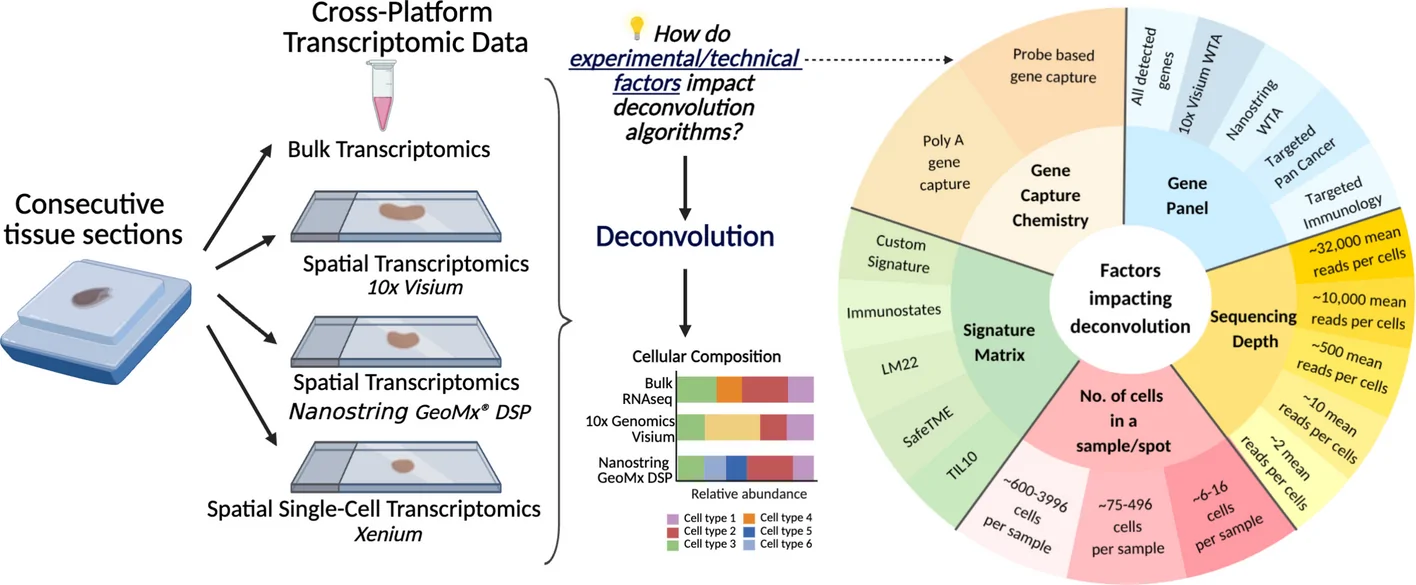

**Supplementary Information:**

The online version contains supplementary material available at 10.1186/s13059-026-04222-8.

## Background

With rapid technological advances, researchers can now quantify gene expression in biological samples using a wide range of transcriptomic profiling platforms. These include bulk transcriptomics from homogenised tissue, single-cell transcriptomics (e.g., 10 × Genomics Chromium, BD Rhapsody), spatial transcriptomics (e.g., spot-based 10 × Genomics Visium, region-based NanoString GeoMx DSP), and spatial single-cell transcriptomics (e.g., 10 × Genomics Xenium, NanoString CosMx). These platforms differ in resolution, measurement technology (sequencing- or imaging-based), mRNA capture chemistry, tissue processing requirements, number of target genes and number of cells profiled. Despite these differences, all of them generate a count matrix that quantifies the gene expression across samples, spots or individual cells. In addition to random measurement errors between technical replicates generated using the same technology, platform-specific technological differences can introduce systematic measurement biases. Both of these effects are commonly referred to as “batch effects”.

To balance cost with measurement detail and to address diverse biological questions, studies increasingly integrate transcriptomic profiles across multiple platforms [1–4]. For instance, a single tissue specimen may be analysed using different platforms, such as bulk transcriptomic and region-based spatial transcriptomics, to gain complementary information. In other cases, publicly available datasets produced using different transcriptomic profiling methods are integrated to investigate specific tissue type or disease condition. Longitudinal samples may be profiled using the same technology but with different chemistries or updated kit versions. Although such scenarios are increasingly frequent, there is currently little consensus on how to consistently and reliably compare transcriptional profiles and downstream analyses across different transcriptomic platforms.

Transcriptional cell-type deconvolution is a common downstream analysis method for datasets without single-cell resolution. It computationally separates a bulk gene expression matrix into its constituent cell types. This approach is particularly valuable for estimating immune cell composition within complex tissues, with applications in oncology and immunology [5–7]. Table 1 lists commonly used computational tools for estimating cell type abundances across different transcriptomic data types.

**Table 1:** Overview of transcriptional deconvolution methods

Tool	Designed for	Mathematical model	Cell-type reference
‍MCPcounter^*^ [8]	signature quantification, bulk	log-sum of marker gene expression	list of marker genes
‍CIBERSORT [9]	deconvolution, bulk	support vector regression	customisable signature reference
‍EPIC [10]	deconvolution, bulk	constrained least squares regression	customisable signature reference
quanTIseq [11]	deconvolution, bulk	constrained least squares regression	customisable signature reference
‍SPOTlight [12]	deconvolution, spot-based spatial	seeded non-negative matrix factorisation (NMF)	requires a mandatory single-cell dataset to build reference
Celloscope [13]	deconvolution, spot-based spatial	Bayesian probabilistic graphical model	customisable signature reference
‍CARD [14]	deconvolution, spot-based spatial	conditional autoregressive model-based deconvolution	requires a mandatory single-cell dataset to build reference
cell2location [15]	deconvolution, spot-based spatial	Bayesian mixture model	requires a mandatory single-cell dataset to build reference
‍SpatialDecon [16]	deconvolution, region-based spatial	log-normal regression	customisable signature reference
RCTD [17]	deconvolution, spot-based spatial	maximum maximum-likelihood cell type proportions	requires a mandatory single-cell dataset to build reference

^*^MCPcounter outputs are most suited for comparing a given cell type across samples [8], but not for comparing different cell types within a sample. In this study, scores were scaled to the interval [0,1] to generate relative cell-type specific value that preserves across-sample distributions (see Methods)

Multiple benchmarking studies have evaluated transcriptional deconvolution tools for both bulk [18–23] and spatial data [24–26], focusing primarily on metrics such as accuracy and computational efficiency. However, most benchmarks have not considered how varying experimental conditions and technological differences across transcriptomic platforms influence the performance and reliability of deconvolution algorithms.

In this study, we evaluate whether deconvolution tools produce reliable and reproducible results across different transcriptomic platforms and experimental conditions. To address this, we apply multiple deconvolution methods to real-world cross-platform transcriptomic datasets. Additionally, we generate simulated datasets to disentangle the effects of specific technological factors. We systematically investigate how the following experimental factors, known to differ across transcriptomic platforms, affect deconvolution outcomes: (i) Gene capture chemistry: The underlying technological principle used to measure gene expression. For example, 10 × Visium v1 3’ captures transcripts through poly-A tail binding, whereas 10 × Visium v2 and the Nanostring GeoMx DSP relies on barcoded probes that hybridise to target mRNA. (ii) Gene panel: The specific set of genes covered by a transcriptomic platform. The gene panel may cover the whole transcriptome (whole transcriptome assay, WTA) or a pre-defined subset of genes (e.g., 10 × Genomics Visium Targeted Pan Cancer and Targeted Immunology Panels). (iii) Sequencing depth: The number of sequencing reads measured per cell in the region of interest or sample. This measure is particularly relevant for WTAs, since the sequencing depth also determines the number of genes detected. (iv) Number of cells measured: The cellular resolution of the assay. Bulk transcriptomics cover about 10^3^–10^6^ cells, whereas region-based spatial methods such as Nanostring GeoMx region of interest (ROI) covers about 10^2^–10^3^ cells [27] and spot based spatial methods such as 10 × Visium (v2, v1 3’) only cover approximately 10 cells per spot [28].

Our results highlight how the different mathematical approaches used in the deconvolution tools are affected by data properties that depend on technological differences between transcriptomic platforms. Moreover, we provide practical guidelines for selecting computational methods dependent on experimental design for robust deconvolution of cross-platform transcriptomic data.

## Results

### Transcriptional deconvolution results vary across transcriptomic platforms and deconvolution tools

**Fig. 1:**
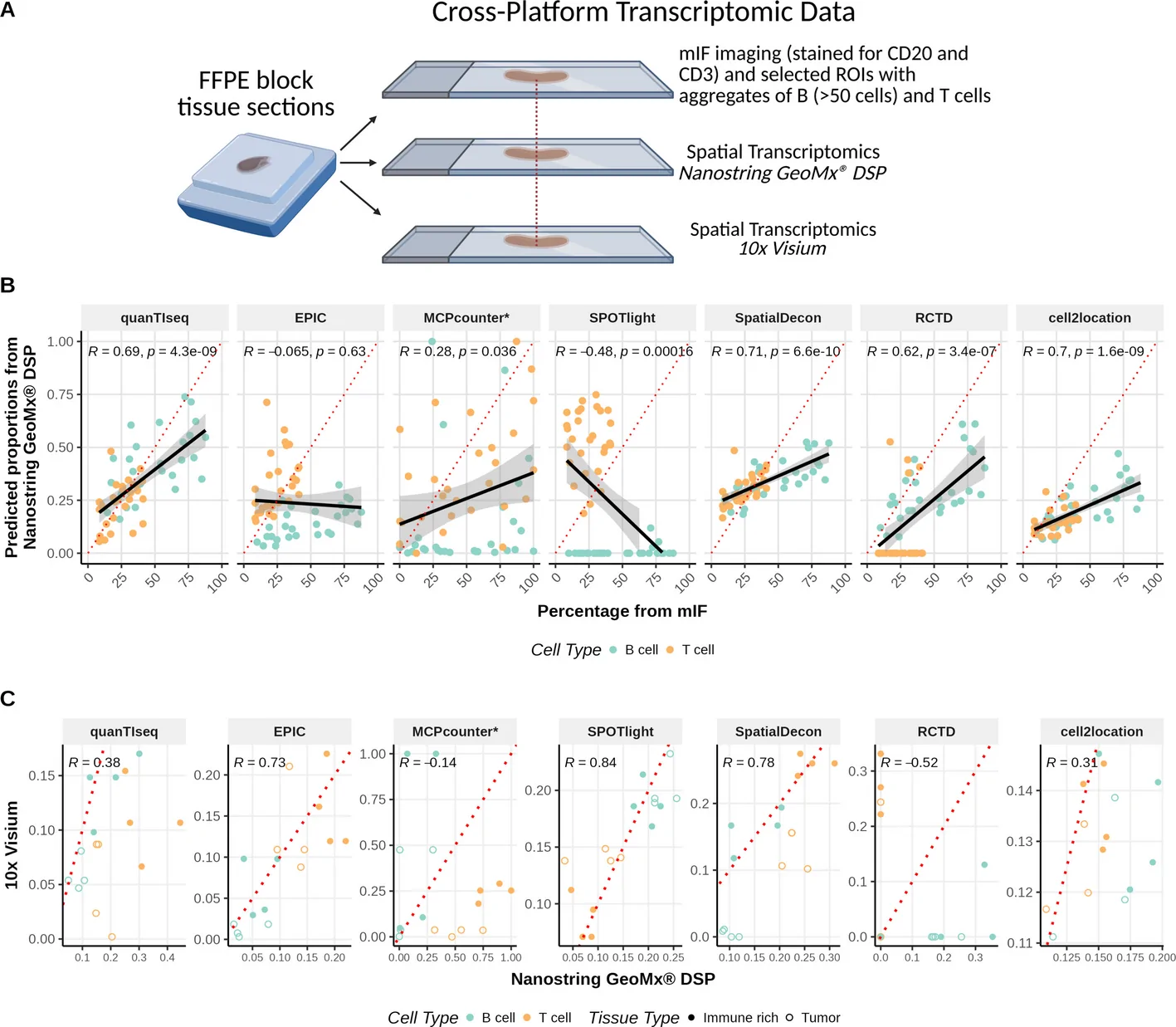
Quantification of B and T cells in cross-platform transcriptomic data. **A** Schematic representation of matched regions of an FFPE tissue block profiled by mIF (stained for CD20 and CD3), Nanostring GeoMx DSP, and 10 × Genomics Visium v2 FFPE spatial transcriptomics. **B** PCC between proportions of B and T cells as seen in mIF images (stained for CD20 and CD3) and the predicted proportions of the cells in the consecutive tissue section that was processed using Nanostring GeoMx DSP. The red dotted line represents the line of identity (x = y). *MCPcounter scores were scaled to [0,1] to create relative cell-type values while preserving their distribution (see Methods). **C** Predicted proportions of B and T cells from immune-rich and tumour-rich regions, derived from 10 × Visium spots and NanoString GeoMx DSP ROIs across four patients. Aggregated data from approximately 50–100 10 × Genomics Visium spots in regions designated as immune-rich or tumour-rich in 10 × Visium are compared against data from 1–4 ROIs per patient in NanoString GeoMx DSP. Colour indicates cell type. Panel A was created with BioRender

Many recent biological studies integrate transcriptomic data from multiple technological platforms to address different biological questions, to balance cost against resolution, or to leverage existing public datasets. To illustrate the challenges of cross-platform transcriptomics and their impact on transcriptional deconvolution and biological interpretation, we reanalysed a dataset from one of our previous studies [1] in which consecutive tissue sections were profiled using different omic methods. The dataset consisted of following measurements focusing on immune aggregates (Fig. 1A): (i) B and T cell counts obtained from multiplex immunofluorescence (mIF, 10 formalin-fixed paraffin-embedded (FFPE) blocks) [1], (ii) transcriptome profiles of 28 immune aggregates and corresponding tumour-rich areas measured using the Nanostring GeoMx DSP platform (WTA gene panel), (iii) transcriptome profiles measured using 10 × Genomics Visium v2 FFPE WTA assay.

We first compared frequencies of B and T cells extracted from mIF with those predicted by deconvolution tools applied to the GeoMx DSP data (Fig. 1B). Deconvolution was performed using quanTIseq [11], EPIC [10], MCPcounter [8], SPOTlight [12], SpatialDecon [16], RCTD [17] and cell2location [15]. These methods represent a subset of deconvolution methods that reflect a broad range of design choices and underlying mathematical models (Table 1). We initially used default signatures for methods that provide them (quanTIseq, EPIC, MCP-counter, SpatialDecon) and a peripheral blood mononuclear cell (PBMC) single-cell dataset (Dataset D listed in Additional file 1: Table S1) as reference for methods requiring scRNA-seq input (SPOTlight, RCTD, cell2location). This reflects common user practice and provides a pragmatic baseline for cross-platform comparisons. Immune aggregates from mIF and Nanostring GeoMx areas could directly be mapped to each other, allowing direct comparison of immune cell frequencies between transcriptomics and proteomics assays. Across 28 ROIs with matched mIF data the overall correlations were moderate and varied across tools. SpatialDecon consistently achieved the highest concordance (Pearson correlation coefficient (PCC) = 0.71) between predicted and observed B and T cell proportions, followed closely by cell2location (PCC = 0.7) and quanTIseq (PCC = 0.69).

Next, we compared B and T cell proportions predicted from GeoMx ROIs and Visium spots (Fig. 1C). In this analysis, we were not able to directly match the ROIs across assays due to considerable histomorphological differences between the consecutive tissue sections. However, we were able to combine data from all immune-rich (delineated manually using B and T cell marker genes) and all tumour-rich regions originating from the same tissue block. Although the cellular composition of immune aggregates is expected to be comparable within a given patient [1], predicted proportions differed substantially between the two platforms, with the extent of difference in predictions depending on the deconvolution method used (Fig. 1C). The best correlation of predictions between both datasets was achieved using a least-square regression (EPIC) and a log norm regression method (SpatialDecon). These methods also correctly predicted higher B and T cell proportions in the immune-infiltrated regions compared to the tumour-rich areas (Fig. 1C).

### Gene panel selection impacts deconvolution results in simulated and experimental data

Given the large variability in deconvolution results across computational methods and technological platforms, we next investigated the impact of individual experimental factors that differ between technologies. We began by examining the effect of the gene panel, i.e. the set of genes measured in a transcriptomic assay. Pre-designed targeted gene panels, which capture only a fraction of the whole transcriptome, are often used to balance cost and technological constraints while capturing the most informative genes [29–31]. Since gene panels vary widely in size and gene selection, they are expected to influence deconvolution outcomes, with the magnitude of this effect likely depending on the method used.

**Fig. 2:**
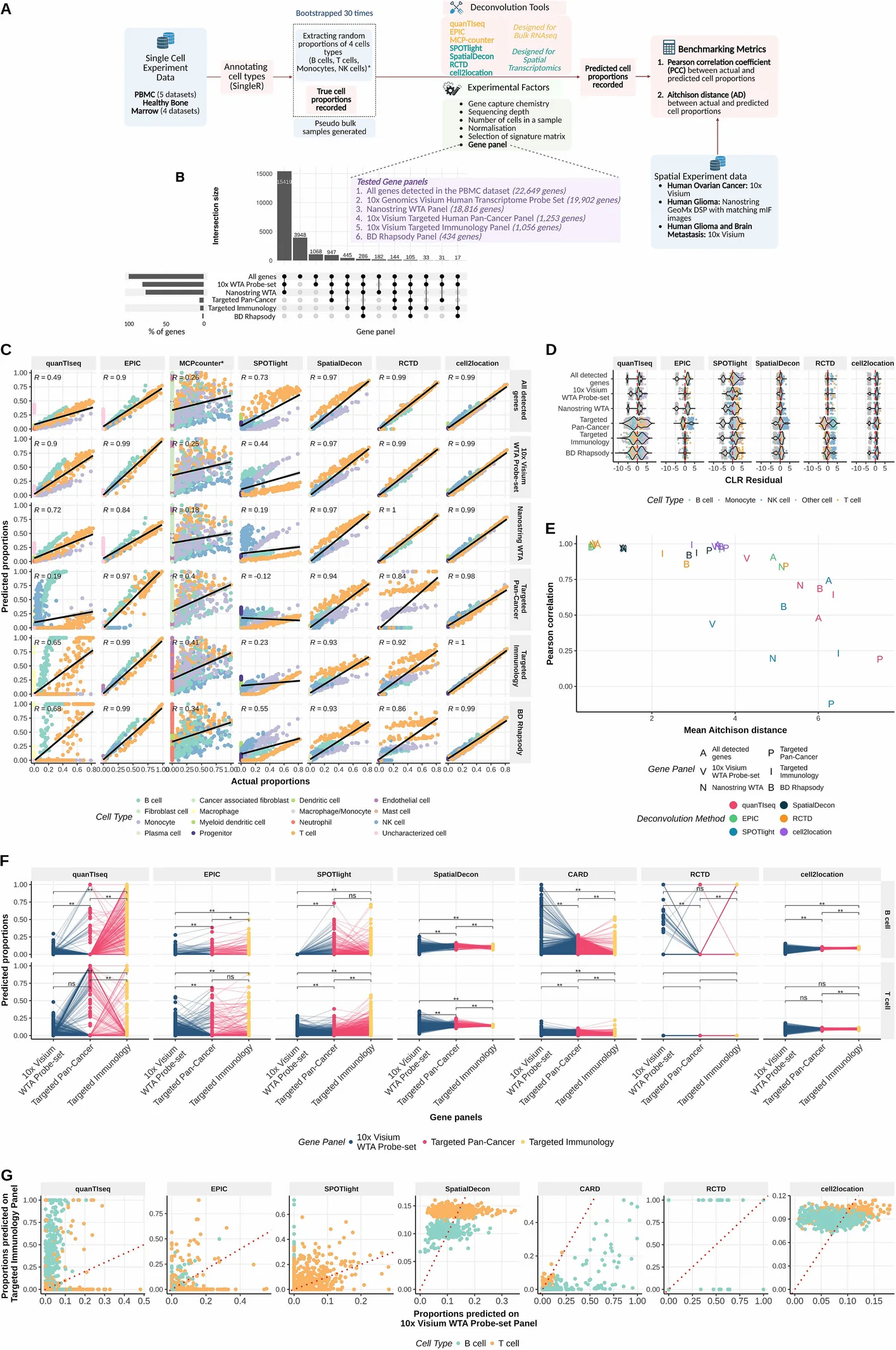
Impact of gene panel on deconvolution performance.** A** Schematic representation of the approach used to simulate pseudo-bulk samples from publicly available PBMC and bone marrow single cell datasets. When testing a particular experimental factor, all other factors were kept constant using their default settings. In this analysis, we tested five commonly used gene panels across seven deconvolution tools. **B** Upset plot showing gene distribution across five gene panels. Three panels (all genes, 10 × Genomics, and Nanostring WTA panel), are large, each containing over 15,000 genes. In contrast, BD Rhapsody panel and the 10 × Genomics targeted panels are much smaller, each representing less than 10% of the total number of genes. **C** Correlation plots comparing actual and predicted cell type proportions in PBMC pseudo-bulk samples. Points are coloured according cell types predicted by each method (Additional file 1: Table S2). Pseudo-bulk samples used as a basis for deconvolution only contained B cells, T cells, monocytes and NK cells. Each plot facet displays R, which is the PCC. **D** Distribution of CLR residuals across deconvolution methods and gene panels. The colour of the dots denotes the cell type. **E** Performance relationship between PCC and mean AD across gene panels on the deconvolution tools in the PBMC pseudo-bulk samples.** F** Predicted proportions of B and T cells across 500 randomly selected spatial spots from the same tissue section in the human ovarian cancer dataset (10 × Visium), evaluated across three gene panels. Proportions range from 0 to 1. Pairwise Wilcoxon tests were performed between panels, with Bonferroni correction applied. Asterisks indicate significance: p = 3.30 × 10⁻^2^ for the comparison between the targeted immunology and targeted pan-cancer panels using EPIC. All other comparisons marked with “**” have p < 8.58 × 10⁻^5^. **G** Predicted B and T cell proportions for 500 randomly selected spatial spots in the human ovarian cancer dataset, comparing the targeted immunology panel to the 10 × Visium WTA panel. Each dot represents the same spot analysed with both panels. Coloured dots represent B cells and T cells. The red dotted line represents the line of identity (x = y). Panel A was created with BioRender

To comprehensively evaluate the choice of the gene panel on the deconvolution results, a systematic data simulation approach was implemented (Fig. 2A). For this, we used single-cell RNA-seq datasets of PBMCs (Dataset A, B, C and D listed in Additional file 1: Table S1) and healthy bone marrow (Dataset E, F and G listed in Additional file 1: Table S1). We annotated various immune cell types, including B cells, T cells, monocytes, and NK cells, dendritic cells, basophils, neutrophils and progenitors using the Monaco Immune Data [32, 33] as a reference and the SingleR package [34] for automated cell-type classification (Additional file 1: Table S2). We then created pseudo-bulk samples by aggregating counts from B cells, T cells, monocytes, and NK cells that were randomly sampled from the annotated cell pool to generate a pseudo-bulk-level count matrix. Each pseudo-bulk sample contained between 200 and 7000 cells in total and per condition 30 pseudo-bulk samples were generated (see also Additional file 1: Table S4).

The genes in the pseudo-bulk-level count matrix were filtered to match four transcriptomic panels: BD Rhapsody Immune Response (in-house, 434 genes) [35], targeted immunology [36] (1,056 genes), targeted pan-cancer [37] (1,253 genes), Nanostring GeoMx WTA [38] (18,816) and 10 × Visium Human Transcriptome [39] (19,902 genes). In addition, we included a panel containing all genes detected in the dataset with existing HUGO gene symbol that were detected in the dataset (22,649 genes in PBMC and 33,514 genes in healthy bone marrow; see Fig. 2B). A full list of genes included in each gene panel is provided in Additional file 1: Table S3. We applied seven deconvolution tools: quanTIseq, EPIC, MCPcounter and SpatialDecon using their default signature matrices, as well as SPOTlight, RCTD and cell2location with dataset D (Additional file 1: Table S1) as signature reference. Predicted cell proportions were then compared to the ground truth across all simulated pseudo-bulk datasets.

We first evaluated deconvolution performance using PCC between the predicted and actual cell proportions (Fig. 2C). However, compositional data such as cell-type proportions are susceptible to spurious correlations [40]. To address this, we used the residuals from the centred log-ratio (CLR) transformation to quantify the differences between actual and predicted cell-type compositions in a pseudo-bulk sample [41] (Fig. 2D). Based on these CLR residuals, we calculated the Aitchison distance (AD) for each sample, which measures the dissimilarity between the compositional vectors of actual and predicted cell type proportion [42] (Fig. 2E) (see Methods for details). cell2location performed most consistently, with little to no differences observed between the different gene panels (Fig. 2C to E). Across all gene panels and for both PBMC and bone marrow datasets, EPIC and RCTD followed by SpatialDecon achieved the best performance showing the highest PCC, the lowest absolute CLR residuals and lowest mean AD (Fig. 2C to E, Additional file 2: Fig. S1). Because the default implementation of EPIC does not support monocyte prediction, monocytes were excluded from all EPIC-related analyses, including simulated data generation. EPIC’s superior performance relative to other tools persisted when all methods were evaluated on monocyte-free pseudo-bulks (data not shown).

The two constrained least square regression methods, quanTIseq and EPIC performed well on large panels. Both tools performed best with the 10 × Visium WTA panel, and less well with the Nanostring WTA panel and the panel consisting of all detected genes (Fig. 2C, D and Additional file 2: Fig. S1A, B). This decrease in performance was mainly due to the prediction of a high proportion of “uncharacterized cells”, which are a result of gene expression that cannot be explained by a function of the signature matrix. This highlights that the prediction of uncharacterised cells by constrained least square regression methods is sensitive to gene panel selection. Indeed, other methods that performed well on large panels (SpatialDecon, RCTD, cell2location) did not present these issues.

The performance of quanTIseq and RCTD decreased for smaller gene panels (Fig. 2C-E). EPIC failed to predict NK cells for the targeted pan-cancer gene panel (Fig. 2C, D, Additional file 2: Fig. S1A-C). The differences observed in the performance of the two least square regression based methods (quanTIseq and EPIC) likely stem from the different signature matrices used in both tools. In particular, quanTIseq had minimal overlap with some panels, e.g. only 23 genes are common between the targeted pan-cancer and the default quanTIseq signature matrix.

SPOTlight showed poor performance with PCC values less than 0.55 (Fig. 1D), and negative values of PCC observed for some gene panels in both PBMC and bone marrow samples (Fig. 2C, Additional file 2: Fig. S1A). The tool tends to overestimate the proportions of progenitors and dendritic cells in the larger gene panels, at the expense of monocytes. Similarly in the smaller gene panels, it also misattributed proportions of monocytes and T cells (Fig. 2D and Additional file 2: Fig. S1).

The comparisons of MCPcounter results with all other tools are more challenging, as it is based on gene signature quantification. To enable comparison, we scaled both the actual proportions and MCPcounter scores to the interval [0,1] for each cell type, thereby preserving the relative distribution across samples (for details see Methods). As a result, the normalised scores did not preserve compositionality within a sample and we could therefore not calculate CLR residuals and AD for MCP-counter. MCPcounter failed to detect small differences in cell type abundance across samples (Fig. 1C).

We next aimed to validate the differences in deconvolution performance across tools and gene panels using real-world cross-platform transcriptomic data. For this, we analysed publicly available 10 × Visium spatial transcriptomic data from the same section of human ovarian cancer tissue, for which three different sequencing libraries covering different gene panels were prepared: whole transcriptome (Dataset WTA), targeted immunology (Dataset TI; 1,056 genes), and targeted pan-cancer panel (Dataset TPC; 1,253 genes) (see Additional file 1: Table S1). We applied seven deconvolution methods (quanTIseq, EPIC, SPOTlight, SpatialDecon, CARD, RCTD and cell2location) and focused on the estimated B and T cell proportions across 500 spatial spots (Fig. 2F). Predicted cell proportions for quanTIseq, EPIC, SPOTlight and CARD varied notably for the same spot, particularly as gene panel size decreased (Fig. 2F, G). For example, quanTIseq predicted significantly higher B and T cell proportions with the targeted immunology panel than the other two panels (Fig. 2F, G), which is also reflective of the observations made with simulated data. Despite EPIC’s good performance on simulated data, the tool did not achieve notable correlation between predictions on WTA and targeted immunology panel in the experimental data (Fig. 2G). SpatialDecon and cell2location showed the greatest variation in B and T cell predictions across spots with the WTA panel, but this variation was nearly absent with the targeted immunology panel (Fig. 2F, G). This decreased variance in predicted values with decreasing gene panel size was also observed in the deconvolution of simulated data for SpatialDecon. With decreasing gene panel size on real-world experimental data, in particular quanTIseq tended to overestimate variability between samples, whereas SpatialDecon and cell2location underestimate this variability.

### Commonly used sequencing depths have minimal effect on deconvolution tools

**Fig. 3:**
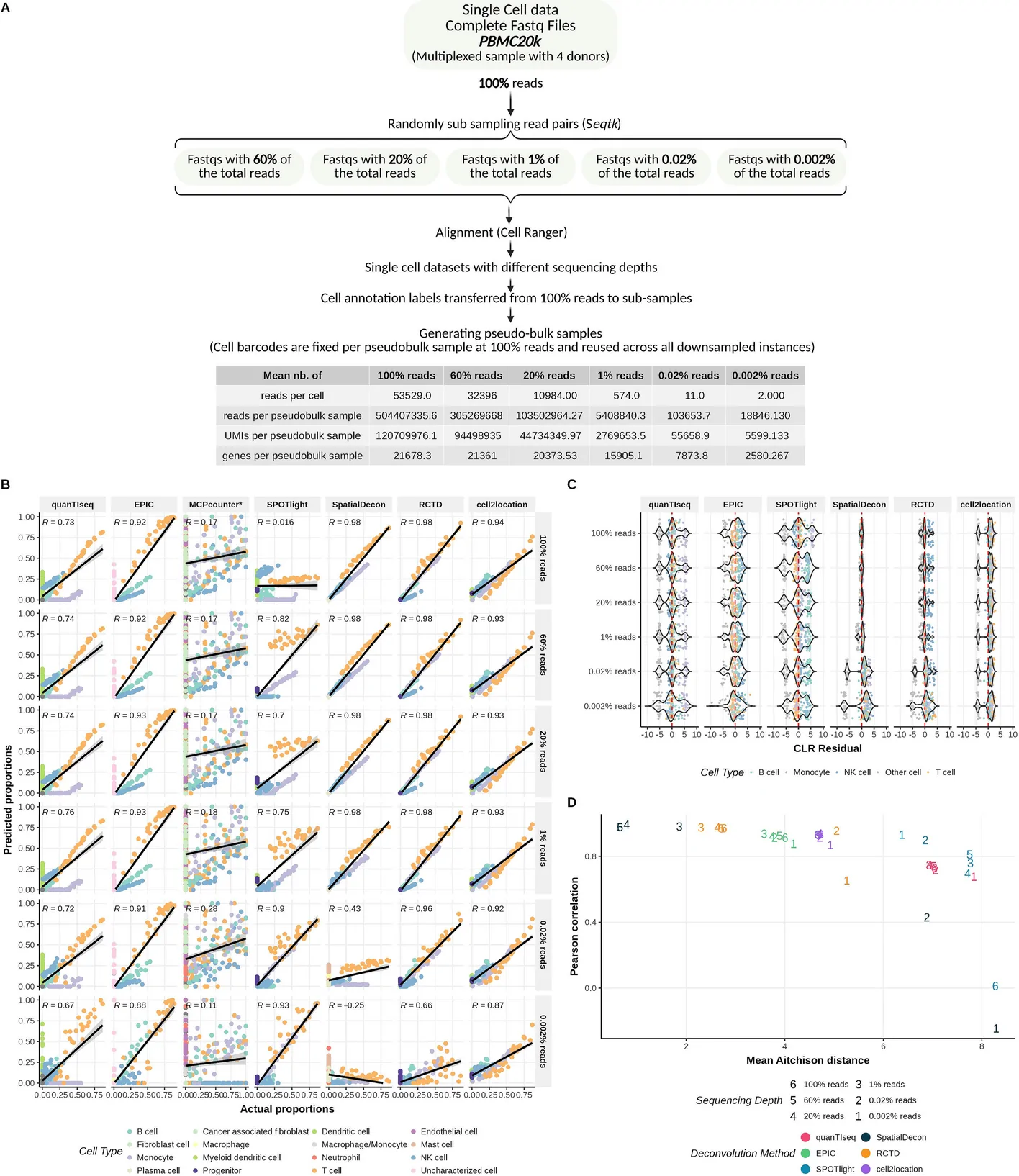
Impact of sequencing depth the deconvolution performance in PBMC pseudo-bulk samples.** A** Reads were randomly downsampled from FASTQ files to obtain 100%, 60%, 20%, 1%, and 0.02% and 0.002% of the original read depth. For each subsample, CellRanger was re-run and cell annotations were transferred from the full dataset (100% reads). Pseudo-bulk samples were generated using the workflow described in Fig. 1B. **B** Correlation between actual and predicted cell type proportions in PBMC pseudo-bulk samples across sequencing depths. Points are coloured according cell types predicted by each method (Additional file 1: Table S2). Pseudo-bulk samples used as a basis for deconvolution only contained B cells, T cells, monocytes and NK cells. PCC is shown in each plot. **C** Distribution of the CLR residuals for each deconvolution method at different sequencing depths. The colour of the dots denotes the cell type. **D** PCC versus mean AD for the performance across sequencing depths and deconvolution methods. Panel A was created with BioRender

Another important experimental factor that affects transcriptomic profiles across technological platforms and experimental designs is sequencing depth, i.e. the number of sequencing reads per cell. To evaluate how the average number of reads per cell affects deconvolution accuracy, we randomly downsampled reads from FASTQ files of two single-cell datasets. PBMC (Dataset H; Additional file 1: Table S1, Fig. 3) and bone marrow (Dataset I; Additional file 1: Table S1, Additional file 2: Fig. S2) datasets were downsampled to 60%, 20%, 1%, 0.02% and 0.002% of the original reads (Fig. 3A and Additional file 2: Fig. S2). Cell Ranger [43] 9.0.0 was rerun for each subsampled dataset to generate feature-barcode matrices. Cells were first annotated and subsequently grouped into pseudo-bulk samples using the full dataset (100% reads). For datasets with reduced sequencing depth (60%, 20%, 1%, 0.02%, and 0.002%), pseudo-bulk samples were constructed by aggregating reads from cellular barcodes corresponding to the cells sampled in the full dataset.

Contrary to expectations, the PCC did not decrease with reduced sequencing depth and the distribution of CLR residuals and AD remained stable across sequencing depths down to 1% of total reads for all deconvolution methods (Fig. 3B to D, Additional file 2: Fig. S2). The performance of SPOTlight increased with lower sequencing depth (Fig. 3B). While SpatialDecon performance declined below 1% sequencing depth (~ 574 reads/cell for PBMC, Fig. 2A; ~ 399 reads/cell for bone marrow, Additional file 2: Fig. S2A), EPIC and cell2location showed minimal deterioration even at 0.02% sequencing depth of (~ 11 reads/cell for PMBC, Fig. 3A; ~ 9 reads/cell for bone marrow, Additional file 2: Fig. S2A). These findings suggest that sequencing depth can be reduced substantially, to a few hundreds of reads per cell, without significantly affecting deconvolution accuracy, offering a potential cost-saving strategy. EPIC and cell2location delivered the most reliable results in samples with low sequencing depth (Fig. 3B to D).

### Impact of the number of cells per sample or spot on deconvolution performance

Transcriptomic platforms capture gene expression at varying cellular resolutions, ranging from spot-based spatial transcriptomics (5–18 cells per spot), to region-based spatial transcriptomics (hundreds to thousands of cells per region), up to bulk transcriptomics (thousands to millions of cells per sample). Accordingly, deconvolution tools have been developed with different assumptions regarding the number of cells per measurement. For example, SPOTlight, RCTD and cell2location were developed for small spatial transcriptomic spots and leverage information across multiple spots, whereas SpatialDecon was designed for GeoMx DSP regions that typically contain hundreds of cells. In contrast, bulk-oriented tools such as quanTIseq, EPIC, and MCP-counter were developed for samples containing thousands to millions of cells.

**Fig. 4:**
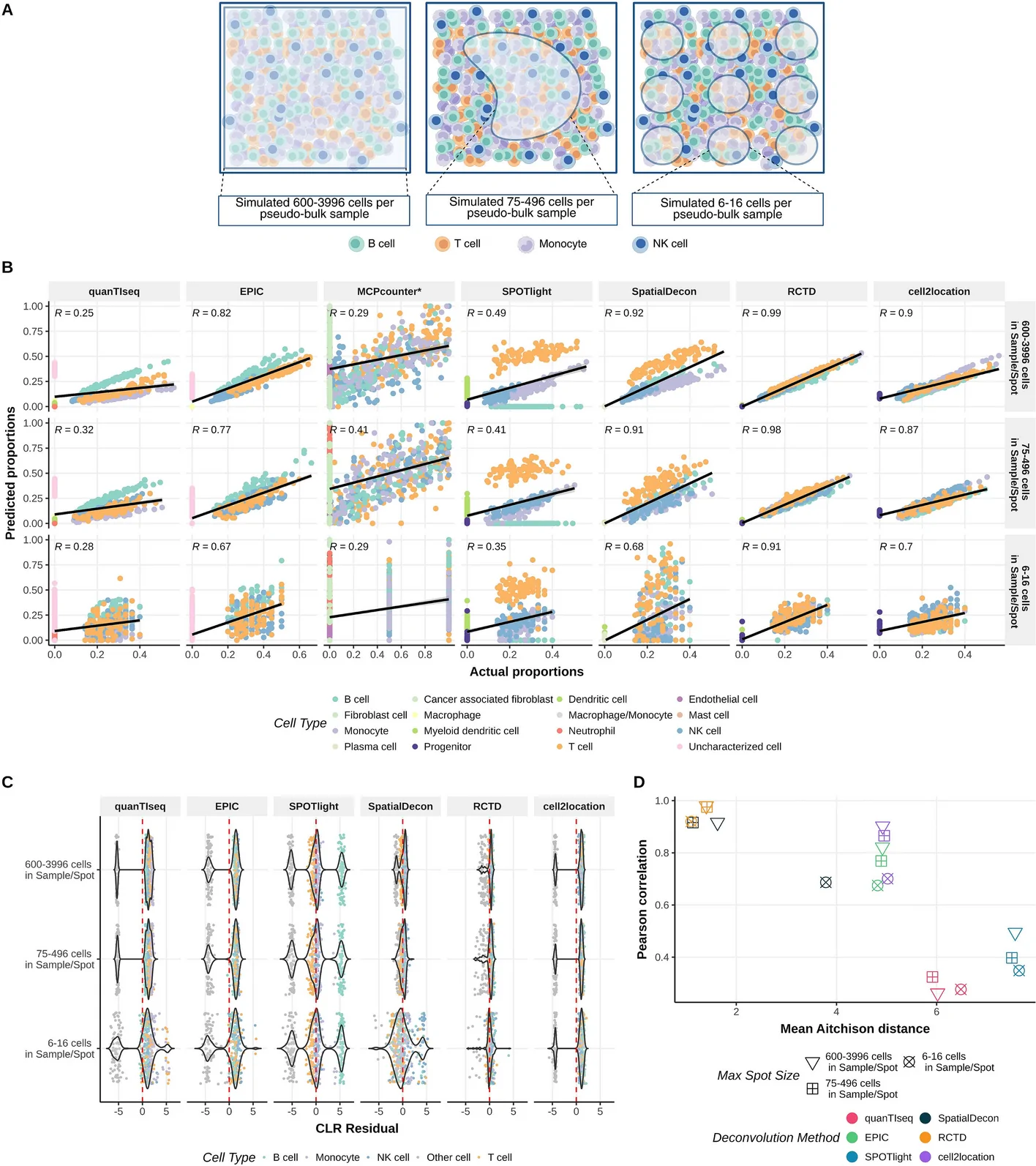
Impact of the total cell number on deconvolution performance. **A** Schematic representations of simulating pseudo-bulk samples or spots of varying sizes. Total cell numbers ranged between 600–3996, 75–496 and 6–16 cells, resembling bulk RNA-seq, Nanostring GeoMx DSP region of interest (ROIs) and 10 × Visium spatial transcriptomic spots, respectively. **B** Correlations between actual and predicted cell type proportions in PBMC pseudobulk samples. Points are coloured according cell types predicted by each method (Additional file 1: Table S2). Pseudo-bulk samples used as a basis for deconvolution only contained B cells, T cells, monocytes and NK cells. The PCC, R, is shown in each facet. *MCPcounter scores were scaled to [0,1] to create relative cell-type values while preserving their distribution (see Methods). **C** Distribution of the CLR residuals for the four cell types included in the PBMC pseudo-bulk samples. Each dot represents a cell type and is colour-coded. **D** PCC versus mean AD, summarising the performance of each deconvolution tool across sample sizes. Panel A was created with BioRender

In our previous simulations, we used an average of ~ 2,000 cells per sample in the PBMC dataset and ~ 600 cells per sample in healthy bone marrow datasets (Additional file 1: Table S4), mimicking region-based or bulk-like conditions. To systematically assess how input cell number affects deconvolution performance, we simulated pseudo-bulk samples composed of four (except EPIC, where we simulated only 3 cell-types) cell types at varying total cell counts: 600–3996 cells per sample (200–999 of each type), 75–496 cells per sample (25–124 of each type), and as few as 6–16 cells per sample (2–4 of each type) (Fig. 4A). Because of these constraints, the true proportion of any individual cell type ranged from 0.06 to 0.7 in these simulations. The sequencing depth (reads per cell) in the pseudo-bulk samples was kept constant across varying cell numbers.

Across all settings, SpatialDecon and RCTD demonstrated the highest accuracy with strong correlation and low AD, followed by EPIC and cell2location (Fig. 4B-D). The performance of RCTD and cell2location was least affected by reducing the number of cells in a sample, making them suitable to deconvolute spot-like sequencing data or compare deconvolution results across samples with varying cell numbers (Fig. 4B-D).

### Gene count normalisation does not affect previous deconvolution results

Normalisation is a standard preprocessing step to account for differences in sequencing depth or sample size. In our previous analyses, we applied TPM normalisation (similar to library-size normalisation) prior to all deconvolution approaches, independent of a tool’s documentation that recommended an alternative strategy. To ensure this choice did not bias our results, we tested each deconvolution method in combination with three commonly used normalisation strategies: TPM normalisation, Q3 normalisation, and quantile normalisation (Additional file 2: Fig. S3A). Across both the PBMC and bone marrow datasets, RCTD and SpatialDecon results produced consistently high PCC and low AD results across all normalisation approaches (Additional file 2: Fig. S3B, C, E, F). Although both RCTD and cell2location are designed to operate on raw counts (without normalisation), both showed marginally improved performance with TPM-normalised input. Overall, normalisation only had marginal influence on performance within each tool, and importantly, the use of TPM normalisation in our prior analyses did not negatively affect predictions (Additional file 2: Fig. S3D, G).

### Choice of signature matrix should depend on gene panel and the cells of interest

As a final step in our study, we investigated the impact of the signature matrix, i.e. the cell type–specific gene expression reference used for deconvolution, on deconvolution performance. Signature matrices can be either be pre-defined or custom-built from tissue-specific single-cell data. First-generation deconvolution tools rely on pre-defined references, whereas second generation tools, such as SpatialDecon or CIBERSORT [9], allow the incorporation of custom single-cell references [23]. However, in many cases, single-cell datasets from the same tissue under the same disease or treatment conditions may be unavailable.

**Fig. 5:**
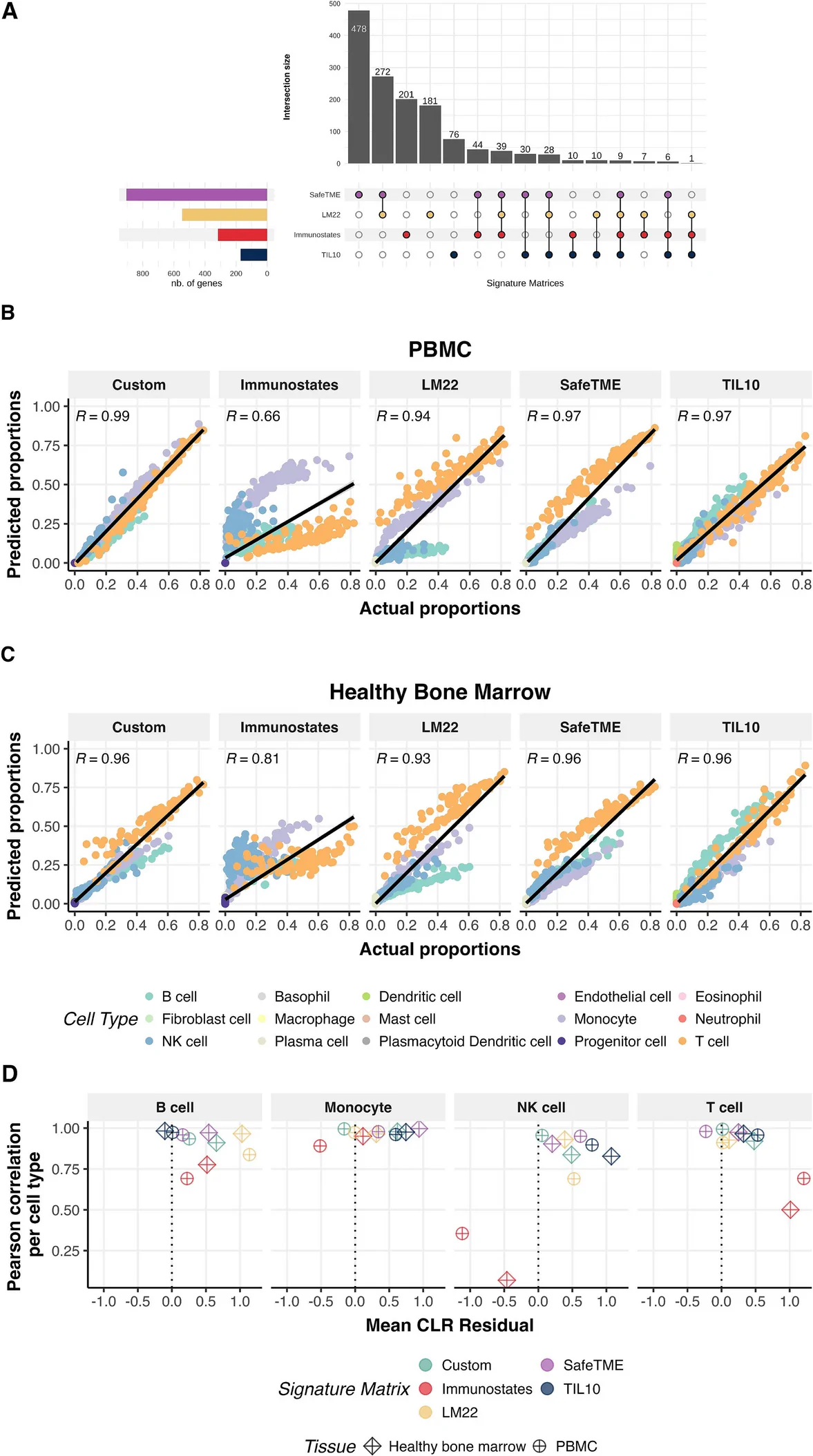
Impact of choice of signature matrix on predicted proportions of cells of interest (using SpatialDecon).** A** Upset plot showing gene overlap among four commonly used immune cell signature matrices: TIL10 (default signature matrix used in quanTIseq), ImmunoStates, LM22 (default signature matrix used with the tool CIBERSORT [9]) and SafeTME (default signature used in SpatialDecon). **B**, **C** Correlations between actual and predicted proportions of cells in the (B) PBMC and (C) bone marrow pseudo-bulk samples. Points are coloured according cell types predicted by SpatialDecon. Pseudo-bulk samples used as a basis for deconvolution only contained B cells, T cells, monocytes and NK cells. **D** Performance evaluation of each signature matrix based on PCC versus mean CLR residuals across four cell types in PBMC and bone marrow pseudo-bulk samples

To assess how well publicly available signature matrices can deconvolute target cell types, we used SpatialDecon, given its robust performance across conditions and support for both built-in and custom matrices (Fig. 5). In addition, we tested the performance of CIBERSORT across different cell signature matrices (Additional file 2: Fig. S4). We evaluated four widely used immune cell signature matrices alongside a custom single-cell–derived reference (Dataset D for PBMC and Dataset F for health bone marrow pseudo-bulk samples). The tested matrices included TIL10 [11], ImmunoStates [44], LM22 [9, 45], and safeTME [16] (Fig. 5A). These signature matrices vary widely in size and gene content, e.g. TIL10 comprises only 170 genes and overlaps with just 9 genes with the others (Fig. 5A).

The safeTME and the single-cell derived custom signature matrix, followed by LM22 and TIL10 matrices showed the best deconvolution performance across gene panels, even when reducing sequencing depth (Fig. 5B, C, Additional file 2: Fig. S5). This suggests that smaller, well curated gene signature can provide comparable results, provided they sufficiently overlap with the gene panel (as seen in quanTIseq performance with smaller gene panel, Fig. 2).

The accuracy of predicted proportions also varied across cell types (Fig. 5D, Additional file 2: Fig. S4). ImmunoStates in combination with SpatialDecon performed better for monocytes and B cells than for NK cells or T cells. These differences emphasize that no single signature matrix is universally optimal; rather, matrix selection should be guided by both the cell types of interest and the biological context of the study. For studies focusing on adaptive immune responses, signature matrices with well-performing B and T cell signatures are suitable (e.g. TIL10 or custom signature matrices).

## Discussion

The rapid development of new transcriptomic platforms and the increasing need to integrate data across technologies underscores the importance of robust deconvolution methods that enable reliable cross-platform comparisons. In this study, we systematically assessed the performance of several deconvolution tools under a range of technological and experimental constraints, using both simulated pseudo-bulk datasets and real-world data from our experiments and publicly available repositories.

Across all analyses on simulated and experimental data, cell2location and SpatialDecon, consistently achieved the most accurate and reproducible results. Their advantage likely stems from the models’ ability to represent the inherent skewness and heteroscedasticity of gene expression data. These methods proved particularly suitable to compare deconvolution results across gene panels and sample sizes, down to cell number of ~ 10 cells per sample for cell2location. Least-squares regression methods such as EPIC and quanTIseq also performed well, but they were more prone to assigning signal to uncharacterised cells. This tendency to predict varying proportions of uncharacterised cells when transcripts from unknown cell types are present may partially explain why EPIC performed considerably better on simulated data than on experimental data.

To assess cross-platform performance, we compared consecutive tissue sections analysed with 10 × Visium v2 and NanoString GeoMx DSP, using mIF-derived cell frequencies as benchmarks. Among all tools, SpatialDecon demonstrated the strongest concordance with experimental data and across platforms. The observed performance variability may be due to differences in mRNA capture technologies: poly(A)-based methods perform best with intact RNA, while probe-based or rRNA depletion-based approaches are preferred for FFPE tissues. These technologies further differ in detection mechanisms (light activation for GeoMx DSP vs. barcoded spatial spots for 10 × Visium), which may introduce systematic biases. As the sections were not spatially registered, heterogeneity in predictions between tissue sections may also contribute to inter-platform discrepancies, which could partially explain the differences between the assays in Fig. 1C.

Using our pseudo-bulk simulation strategy, we further investigated how sequencing depth and number of cells per sample influence deconvolution accuracy. Our data simulation strategy includes simulation of sequencing depth and spot size and expands upon existing pseudo-bulk simulation methodologies proven reliable for benchmarking deconvolution tools [18, 19, 46]. Specifically, we model sequencing depth as the number of reads per cell and use read downsampling to simulate lower sequencing depths, thereby enabling us to disentangle the effects of cell number and sequencing depth. We acknowledge that residual confounding between total library size and cellular mRNA content of a cell cannot be fully eliminated within this framework. We therefore restricted our analysis to cell populations with relatively similar mRNA content, excluding cell types with unusually high transcript abundance.

The simulation of sequencing depth indicated that reliable deconvolution using log-normal or least-square regression is achievable with approximately ~ 300 reads per cell. While this finding covers deconvolution of broad cell types, higher coverage may be necessary for resolving fine cell type definitions. In our study, larger numbers of cells per sample resulted in stable estimates across multiple tools, with best results achieved for SpatialDecon and EPIC. The deconvolution results became less accurate for smaller numbers of cells, and SpatialDecon performance was most affected by the decrease in spot-size. For deconvolution of small cell populations, cell2location and RCTD performed best and therefore represent suitable approaches for also comparing cellular abundances across both small and large samples.

We further investigated how the breadth of gene coverage influences deconvolution performance. While transcriptome-wide measurements are more and more frequent in research context, smaller targeted gene panels remain relevant, particularly in clinical and translational contexts and large patient cohorts [29–31], where cost are an important consideration. cell2location showed stable performance across different gene panels. For other tools, deconvolution accuracy decreased markedly when the overlap between the gene panel and the reference signature matrix was limited, underscoring the importance of gene selection in panel design.

Our gene panel comparisons have broader implications for experimental design. Notably, the widely used TIL10 signature matrix (170 genes) performed well in whole-transcriptome settings but exhibited limited overlap (< 60 genes) with commonly used targeted immunology and pan-cancer panels. These findings suggest that incorporating such non-traditional yet informative genes into targeted panels could substantially improve cell-type resolution, particularly in immune-rich tissues. Designing probes that capture this broader set of informative features may therefore represent a valuable strategy for future spatial and single-cell transcriptomic studies.

Finally, it is important to distinguish between two types of batch effects in transcriptomic data: i) random technical variation may occur between independent measurements generated using the same technology and ii) systematic batch effects arise from differences between measurement technologies or experimental protocols. Our study evaluates the latter and our analyses do not assess the influence of gene-specific random technical variability within a single technology, which would require experimental designs including technical replicates and independent estimation of gene-level technical variance.

## Conclusions

In summary, SpatialDecon demonstrated the most consistent performance across both simulated and experimental conditions, providing a robust framework for accurate cross-platform transcriptomic deconvolution of region-based or bulk data. On the simulated data, cell2location showed robustness to variations in experimental factors, including gene panel size, sequencing depth, and cell number, enabling reliable deconvolution at the spot level. However, this good performance comes at the cost of substantially higher computational requirements compared to SpatialDecon. RCTD also performed well in settings with limited cell numbers, making it a suitable option for small cell populations, but exhibited reduced reliability in cross-platform comparisons using experimental data.

## Methods

### Cross-platform datasets included in the study

Details of all the datasets used in the study are provided in Additional file 1: Table S1.

#### 10 × Visium data from human ovarian cancer (publicly available)

10 × Genomics Visium human ovarian cancer datasets with different gene panels (Dataset WTA [47], TI [48], TPC [49] as listed in Additional file 1: Table S1) were accessed using the TENxVisium package [50] from Bioconductor.

#### Sample collection for in-house datasets (Nanostring and Visium)

Clinical data and tumour samples (FFPE tissue blocks) were obtained upon informed consent of the patients. The study was conducted in accordance with ethical standards such as the Declaration of Helsinki. The study protocol was endorsed by the ethical committee of the University Cancer Center (UCT) Frankfurt (SNO-4–2022 and SNO-3–2025).

#### Nanostring GeoMx DSP from human glioma (generated in-house)

Nanostring GeoMx DSP data from glioma samples (Dataset NG [51, 52], Additional file 1: Table S1), including matching mIF image data (for 28 ROIs from 10 patients), were generated in-house. These datasets, along with their processing, are detailed in Cakmak et al. (2025) [1] and are available under GEO accession ID (GSE271255). Two brain metastasis (Dataset NG_BM [52], Additional file 1: Table S1) samples were also processed using the same protocol from Cakmak et al. (2025) [1], and the data is available under GEO accession ID (GSE338144).

#### 10 × Visium data from human glioma and brain metastasis (generated in-house)

Four samples (2 glioma and 2 brain metastasis) (Dataset VG [53], Additional file 1: Table S1) processed with Nanostring GeoMx DSP platform were also analysed using the 10 × Genomics Visium protocol to enable cross-platform comparison. FFPE tissue Sects. (5 µm thick) were placed onto a Visium Spatial Gene Expression slide and heated at 42 °C for 3 h on a thermocycler using the Visium PCR adaptor. The slides were then processed according to the manufacturer’s protocol for the Visium Spatial Gene Expression for FFPE workflow (CG000160, CG000239, CG000407; 10 × Genomics, California, USA).

Briefly, slides underwent deparaffinisation followed by hematoxylin and eosin (H&E) staining. Stained slides were imaged using the PhenoImager HT (Akoya Biosciences, Massachusetts, USA). After imaging, coverslips were removed, and the slides were subjected to decrosslinking, probe hybridisation, probe ligation, probe extension and library construction. Libraries were sequenced with paired-end dual-indexing (28 cycles Read 1, 10 cycles i7, 10 cycles i5, 90 cycles Read 2) on a NextSeq 1000 sequencer (Illumina, California, USA).

Reads were aligned to the human genome reference GRCh38-2020-A using the Space Ranger 2.0.0 pipeline (10 × Genomics), and gene expression counts were quantified. Immune-rich regions were annotated based on CD20 expression using Loupe Browser (version 6.2, 10 × Genomics). The gene expression data from these samples under GEO accession ID GSE338314. After sequencing, data corresponding to spots within CD20-enriched regions were extracted (approximately 50–100 spots per patient) following visual inspection of CD20 gene expression using the Loupe Browser. These regions were considered immune-rich areas. Tumour-rich areas were defined in regions with high tumour cell density.

#### Deconvoluting visium and nanostring data

For the 10 × Visium datasets (Dataset VG [53], Additional file 1: Table S1), only spatial barcodes classified as ‘immune-rich’ and ‘tumour-rich’ based on CD20 expression were used for comparison with ‘immune-rich’ and ‘tumour-rich’ regions identified by the Nanostring GeoMx DSP (Dataset NG [51, 52], Additional file 1: Table S1) in human glioma and brain metastasis samples. We aggregated gene expression data per patient into the two groups described above, enabling direct comparison of immune status across both platforms. Aggregated data from Visium spot and GeoMx ROIs was normalised using a TPM method. To reduce platform-specific bias, only genes shared between the two platforms were retained for downstream deconvolution analyses.

#### Comparing transcriptional deconvolution of Nanostring data to mIF proteomic cell counts

For comparison of the 28 ROIs from 10 glioma patients (subset of Dataset NG [51, 52], Additional file 1: Table S1), the ‘immune rich regions’ from Nanostring GeoMx DSP was analysed and matched to mIF data. For the immunofluorescence part, 10 glioma FFPE blocks were stained for B cells (CD20) and T cells (CD3). A pathologist annotated aggregates of immune cells and counted the number of B and T cells in each immune aggregate to establish ground truth cell counts from proteomics assay. The corresponding gene expression count matrix was TPM-normalised, and all genes from the Nanostring WTA panel were included for deconvolution using signature matrix based (quanTIseq, EPIC, MCPcounter or SpatialDecon (signature matrix = safeTME, bg = 0.01)) and scRNA-seq reference based (SPOTlight, RCTD, cell2location) tools with a PBMC reference (Dataset D [54], Additional file 1: Table S1).

### Generation of simulated datasets from single-cell transcriptomic data

#### Single-cell sequencing datasets (publicly available)

We analysed several publicly available single-cell RNA-sequencing datasets. Four 10 × Chromium PBMC samples (Datasets [54–57] A, B, C and D; Additional file 1: Table S1) were downloaded using the Bioconductor package TENxPBMCData [58]. Datasets A and C originate from the same patient, while Datasets B and D are from another patient. Three additional 10 × Chromium bone marrow datasets (Datasets [59, 60] E, F, G; Additional file 1: Table S1) from three distinct healthy donors were obtained from Bailur et al. (2020) [59]. All the above-mentioned datasets had already been pre-processed with Cell Ranger [43] in their original studies. Dataset A and C were previously processed on Cell Ranger version 1.1.0 with hg19 transcriptome. Dataset B and D were previously processed on Cell Ranger version 2.1.0 with GRCh38 transcriptome with Single Cell 3’ v2 chemistry. Dataset E, F and G were previously processed on Cell Ranger version 1.2.0 with hg38 transcriptome.

For our sequencing depth analysis, we retrieved the raw FASTQ files of PBMC20k dataset (Dataset H [61]; Additional file 1: Table S1) and 20 K bone marrow dataset (Dataset I [62]; Additional file 1: Table S1), from the 10 × Genomics website.

For scRNA-seq-based deconvolution (SPOTlight, RCTD, CARD, and cell2location) of the 10 × Visium human ovarian cancer data, the single-cell reference was downloaded from CellxGene [63, 64]. From this dataset, 33,065 cells were subset by selecting assay = 10 × 3′ v2 and disease = ovarian cancer (Dataset HOC [52, 63]; Additional file 1: Table S1).

#### Cell-type annotation

Data from each origin (PBMC or bone marrow) were compiled into SingleCellExperiment objects [65]. Preprocessing included the removal of cells identified as outliers based on mitochondrial gene content. Cells were clustered using the k-nearest neighbour graph (k = 10), using the scran package [66]. Cell type annotation was performed using SingleR [34], with the MonacoImmuneDataset [32, 33] from the celldex package [34] as the reference. For consistency in downstream analysis, we grouped fine-grained cell types into broader categories, e.g. CD8 + T cells and CD4 + T cells were labeled as “T cells”. A summary of the cell types identified in single-cell datasets and their grouping into broad cell-type categories is provided in Additional file 1: Table S2.

#### Generation of simulated bulk samples

The sampling of cells for pseudo-bulk samples follows these steps: 1. the number of cells for each cell type is drawn from a set of possible numbers (between minimum 10 cells and maximum the total number of cells from the respective cell type in the reference dataset); 2. for each cell type, this number of cells is drawn from the annotated cells in the single-cell reference (sampling without replacement). For this, T cells were sampled exclusively from the T cell population, B cells from the B cell population, and so forth. To assess the impact of the number of cells per sample (Fig. 4), the range of cell number per cell type and per pseudo-bulk was adjusted accordingly (see also Additional file 1: Table S4). Raw count expression values were aggregated to generate pseudo-bulk samples. The summary of the number of cells that went into the pseudobulks for each result figure are given in Additional file 1: Table S4. To reduce sampling bias and improve statistical robustness, the sampling process was bootstrapped 30 times for each single-cell dataset, creating replicates and capturing intra-cellular heterogeneity in each sample. The actual numbers of cells of each cell type used in each pseudo-bulk sample was recorded during this process.

##### Simulation of different gene panels

We tested five commonly used gene panels using pseudo-bulk data:

- 10 × Visium Human Transcriptome Probe Set (v1.0) [39]: Contains 19,144 genes targeted by 19,902 probes.
- Nanostring GeoMx Human Whole Transcriptome Atlas [38]: Includes 18,816 genes.
- 10 × Visium Targeted Immunology [36]: Contains 1056 genes.
- 10 × Visium Targeted Pan Cancer [37]: Contains 1253 genes.
- BD Rhapsody Immune Response Panel: A custom-designed primer panel was added to the predesigned human Immune Response Targeted Panel (BD Biosciences, cat# 633,750) to amplify the cDNA of 434 different genes. This gene panel is published in Bexte et al., (2024) [35].

As a positive control, a panel containing all genes detected in the dataset (that have HUGO approved nomenclature) was used (22,649 for PBMC and 33,514 for healthy bone marrow). This approach allowed us to evaluate the effect of gene panel choice across commonly used panels. Details of the genes included in these panels are provided in Additional file 1: Table S3.

Pseudo-bulk samples were subset based on the gene panel being tested, and deconvolution pipelines were run to predict cell types. For other parameter tests, the default panel (all genes) was used.

##### Simulation of sequencing depth

Using the “PBMC20k” (Dataset H in Additional file 1: Table S1) and “20 k bone marrow mononuclear cells” (Dataset I in Additional file 1: Table S1) datasets, we simulated reduced sequencing depths by downsampling the original reads to 60%, 20%, 1%, 0.02% and 0.002% using seqtk [67]. The resulting FASTQ files were aligned with Cell Ranger (version 9.0.0) [43], using the GRCh38-2024-A reference genome. Cells in the full dataset (100% reads) were annotated following the protocol described in the ‘Cell-type annotation’ section. These annotations were transferred to the downsampled datasets to ensure reliable and consistent cell type labeling across all sequencing depths, as annotations at very low read depths (e.g., median ~ 2 reads/cell at 0.002%) tend to be unreliable. Cells were first annotated and subsequently grouped into pseudo-bulk samples using the full dataset (100% reads). For datasets with reduced sequencing depth (60%, 20%, 1%, 0.02%, and 0.002%), pseudo-bulk samples were constructed by aggregating reads from cellular barcodes corresponding to the cells sampled in the full dataset.

##### Simulation of different cell numbers per sample

We evaluated three spot sizes to study the effects of sample size on deconvolution:

- 6–16 cells per spot: Mimics 10 × Visium spots, includes 2–4 cells of each cell types tested in the analysis.
- 75–496 cells per spot: Represents Nanostring GeoMx DSP regions, with a minimum of 25 cells and a maximum of 124 cells per cell type.
- 600–3996 cells per spot: Emulates larger tissue sections; containing between 200 and 999 cells per cell type.

For other analyses, we used a minimum of 10 cells and up to the maximum available cells per type in each dataset.

### Transcriptional deconvolution

#### Tools used in this study

We applied nine deconvolution tools to estimate the cell composition of the samples:

- quanTIseq [11]: Uses constrained least-squares regression with TIL10 as the default signature matrix.
- EPIC [10]: Based on least-squares regression and includes BRef and TRef signature matrices. TRef is used as the default. The documentation recommends using mRNAProportions for benchmarking the tool with in-silico generated samples and cellFractions for true bulk samples [10, 68], and we therefore, we used mRNAProportions for analyses of simulated data and cellFractions for real experimental data.
- MCPcounter [8]: Estimates cell-type abundance using the log2 geometric mean of predefined marker gene sets. The output scores are best suited for comparing a given cell type across samples but not directly comparable between cell types within the same sample. Therefore, we scaled the predicted scores for each cell type to the [0,1] interval across samples using the following formula:
  $$\text{Scaled Deconvolution Score}_{i,j}=\frac{\text{Deconvolution Score}_{i,j}\;-\;\min\;(\text{Deconvolution Score}_i)}{\max(\text{Deconvolution Score}_i)-\;\min({\mathrm{Deconvolution\;Score}}_i)}$$
  where *i* is the cell type and *j* is the pseudo-bulk sample. This allowed the preservation of the relative distribution of each cell type across samples. Actual cell counts in the pseudo-bulk samples were scaled using the same procedure to allow meaningful comparison with the predicted values. The above mentioned tools were accessed using the ImmuneDeconv R package [69], which provides a unified interface for multiple bulk RNA-seq deconvolution methods.
- SPOTlight [12]: Uses seeded non-negative matrix factorisation (NMF) regression and requires a single-cell reference to build the signature matrix. For PBMC pseudo-bulk samples, Dataset D was used as the single-cell reference, while Dataset F was used for healthy bone marrow pseudo-bulks.
- SpatialDecon [16]: Employs log-normal regression and includes the SafeTME signature matrix as the default. Except in Fig. 5, SpatialDecon was run with its default SafeTME signature.
- RCTD [17]: It predicts the maximum likelihood of cell type proportions based on a scRNA-seq reference based probabilistic model. For PBMC pseudo-bulk samples, Dataset D (Additional file 1: Table S1) was used as the single-cell reference, while Dataset F (Additional file 1: Table S1) was used for healthy bone marrow pseudo-bulks. The deconvolution was performed using the “full” mode to detect all the cell types present in the scRNA-seq reference. For the deconvoluting the real experimental data from brain tumours, “full” mode was used along with Dataset D signature. Ovarian cancer deconvolution was carried out using a ovarian cancer scRNA-seq reference dataset (Dataset HOC, Additional file 1: Table S1).
- cell2location [15]: Uses a Bayesian probabilistic model to estimate cell type proportions in sequencing based spatial transcriptomics. For PBMC pseudo-bulk samples, Dataset D was used as the single-cell reference, while Dataset F was used for healthy bone marrow pseudo-bulks. For the deconvoluting the real experimental data from brain tumours, “full” mode was used along with Dataset D signature. Ovarian cancer deconvolution was carried out using a ovarian cancer scRNA-seq reference dataset (Dataset HOC, Additional file 1: Table S1). The following parameters were used to while deconvolution: min cells = 2000, regularisation constant = 100 for 10,000 training epochs.
- CARD [14]: It employs a conditional autoregressive model for spatial transcriptomics deconvolution. In addition to a mandatory scRNA-seq reference, it requires the spatial coordinates of each spot or ROI, which limited its applicability in our cross-platform benchmarking analysis. It was therefore applied only to the 10 × Visium human ovarian cancer dataset, where spatial coordinates were available, using an ovarian cancer scRNA-seq reference (Dataset HOC, Additional file 1: Table S1).
- CIBERSORT [9]: It is based on support vector regression and was originally designed for bulk transcriptomic deconvolution. Its default signature matrix is LM22, with an option to generate a custom matrix from scRNA-seq data. However, the tool is not publicly available and requires account registration to access the web platform and a token request to the authors for Docker access. Given this limited accessibility, we used it only to evaluate signature matrix performance. Deconvolution was performed via the CIBERSORTx web platform (https://cibersortx.stanford.edu/), where pseudobulk expression data were uploaded and results downloaded.

Details of the cell types detected by each deconvolution tool are provided in Additional file 1: Table S2.

#### Different normalisation approaches

We evaluated four different normalisation methods for the pseudo-bulk datasets:

- Raw counts: Non normalised gene expression values.
- TPM (Transcripts per million): Gene counts divided by the total count of all genes in the pseudo-bulk sample, scaled to one million.
- Q3 normalisation: Counts normalised by dividing each gene’s value by the 75th percentile (Q3) value within the sample.
- Quantile normalisation: Normalises within rows and columns by ranking genes and calculating a row-wise average per gene.This was performed using the preprocessCore package [70].

All pseudo-bulk datasets were normalised simultaneously under each method, and the deconvolution tools were run to predict cell-type composition. TPM was used as the default normalisation method for all other analyses unless otherwise specified. For RCTD and cell2location, which require raw count input, we applied TPM normalisation followed by rounding to the nearest integer, ensuring consistent input data across all deconvolution methods.

#### Signature matrices

We evaluated four published immune cell signature matrices:

- *ImmunoStates* [44]: Comprises 319 genes representing 20 immune cell types, created using diverse PBMC datasets to capture broad signatures.
- *LM22* [9, 45]: Includes 547 genes for 22 cell types. This matrix is commonly used with the CIBERSORT tool.
- *TIL10* [11]: Consists of 170 genes for 10 cell types. It is the smallest matrix tested and is used by quanTIseq.
- *SafeTME* [16]: Contains 906 genes across 18 cell types. It is the default matrix for SpatialDecon.

In addition to these, we generated custom signature matrices using a reference dataset corresponding to each pseudo-bulk dataset (Dataset D for PBMC, Dataset F for bone marrow; Additional file 1: Table S1). These matrices included all genes from the pseudo-bulk data and cover all identifiable cell types in the reference. SpatialDecon and CIBERSORT were used to test these matrices, as they supports both single-cell and user-defined signatures. Unless otherwise stated, each deconvolution tool was run with its default matrix.

#### Benchmarking metrics

##### Pearson correlation coefficient

Pearson’s R values were calculated between the actual and predicted proportions. Theses were calculated while plotting using the stat_cor() function from the R package ggpubr R package [71] and visualised on correlation plots.

##### Aitchison distance (AD)

Aitchison Distance [40, 41] was used to measure the compositional differences between the actual and predicted cell-type proportions. For a given pseudo-bulk samples, AD was calculated using the following formulas:

$$\begin{array}{c} d_{A} (x, y) = \sqrt{\frac{1}{D} \sum\limits^{D}_{i=1} \mathrm{CLR}\_\mathrm{residual}^{2}_{i}}\\ \mathrm{CLR}\_\mathrm{residual}_{i} = \log \left( \frac{x_{i}}{g(x)} \right) - \log \left( \frac{y_{i}}{g(y)} \right)\\ g(x) = \exp \left( \frac{1}{n} \sum\limits^{n}_{i=1} \log\ x_{i} \right) \quad \mathrm{and} \quad g(y) = \exp \left( \frac{1}{n} \sum\limits^{n}_{i=1} \log\ y_{i} \right) \end{array}$$

where:

- ${d}_{A}$ is Aitchison Distance for a pseudo-bulk sample
- ${x}_{i}$ and ${y}_{i}$ are the actual and predicted proportion of a cell-type in a pseudo-bulk sample
- g(x) is the geometric mean of X (actual proportions) and g(y) is the geometric mean of Y (predicted proportions).
- D is the number of components in a pseudo-bulk sample, i.e. number of cells in a pseudo-bulk sample.

The actual compositions included only four cell types (B cells, T cells, monocytes, NK cells; except for EPIC which excluded monocytes), while some deconvolution tools predicted additional cell types. To address this, all the additional predicted cell types were grouped as ‘Other cells’. This ensured both actual and predicted compositions had the same number of components and preserved the Aitchison geometry.

To handle discrepancies:

- If a cell type was predicted but not present in the actual data, its actual proportions was set to 0.
- If a cell type was present in the actual data but not predicted, its predicted proportion was set to 0
- A small offset (0.001) was added to all proportions to avoid zeros, since the AD operates on CLR transformed values, which require a positive input [42].

Mean AD was calculated by averaging the AD across all pseudo-bulk samples of a specific experimental subfactor.

### Generating plots and figures

All plots were generated using ggplot2 [72] and multi-panel figures were assembled using ggpubr [71]. Figures 1A, 2A, 3A, 4A were created using BioRender. The graphical abstract was created using BioRender and Canva.

## Supplementary Information

Additional file 1: Supplementary Tables.

Additional file 2: Supplementary Figures.

## Data Availability

The datasets supporting the conclusions of this article are available as follows. In-house-generated datasets include both data newly generated for this study and data from our previous study by Cakmak et al. [1]. Nanostring GeoMx data from glioma, originally generated for our previous study1, are available via GEO accession GSE271255 [51]. Two datasets were newly generated for the present study: Nanostring GeoMx data from brain metastasis samples, available via GEO accession GSE338144 [52]; and Visium data from glioma and brain metastasis samples, available via GEO accession GSE338314 [53]. Publicly available third-party datasets used include 10 × Genomics ovarian cancer spatial datasets [47–49], 10 × Genomics PBMC datasets (3 k, 4 k, 6 k, 8 k, 20 k) [54–57, 61] and a bone marrow (20 k) dataset [62], a paediatric bone marrow dataset (GSE154109) [59, 60], and an integrated multi-tissue tumour microenvironment single-cell atlas available via CELLXGENE [63, 64]. Further details on all datasets are provided in the Methods section and Additional file 1: Table S1. All scripts used for data processing, normalisation, deconvolution, benchmarking the mentioned experimental factors and for generating the figures in the manuscript are available in the Github repository [73] with the MIT license: https://github.com/AGImkeller/crossplatform_deconvolution A Zenodo repository [74] containing the scripts, data, intermediate data, results and the scripts for generating and assembling the figures in the manuscript can be found here (Creative Commons Attribution 4.0 International license): https://doi.org/10.5281/zenodo.19677865
