# Supplementary material for: Benchmarking cell-type deconvolution in cross-platform transcriptomic data
Source: Genome Biol. 2026 Aug 13;27:265. doi: 10.1186/s13059-026-04222-8 (PMC13495209; doi:10.1186/s13059-026-04222-8)
Supplement: Supplementary file 1 [file 13059_2026_4222_MOESM2_ESM.pdf]

ADDITIONAL FILE 2: SUPPLEMENTARY FIGURES

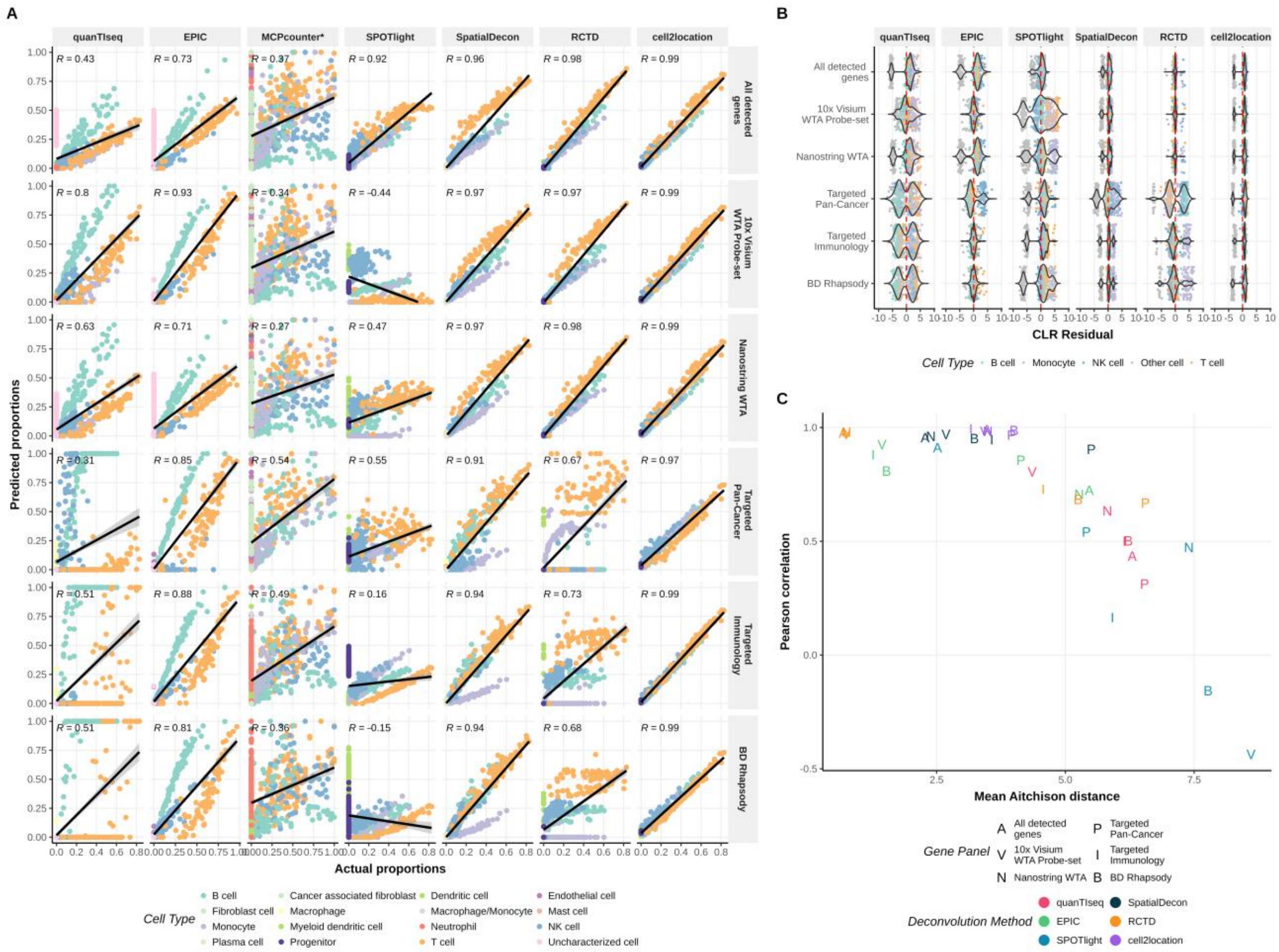

Fig. S1: Impact of gene panel on deconvolution performance in bone marrow pseudo-bulk samples

(A) Correlation plots between actual and predicted proportions of cells in bone marrow pseudo-bulk samples. Points are coloured according cell types predicted by each method (Additional file 1: Table S2). Pseudo-bulk samples used as a basis for deconvolution only contained B cells, T cells, monocytes and NK cells. Each plot facet displays R, which is the PCC.

(B) Distribution of CLR residuals by deconvolution method and gene panel. The colour of the dots denotes the cell type.

(C) PCC versus mean AD for the performance of gene panels on the deconvolution tools in the PBMC pseudo-bulk samples.

A

Mean nb. of	100% reads	60% reads	20% reads	1% reads	0.02% reads	0.002% reads
reads per cell	38316.00	23047.00	7748.0	399.00	9.000	2.000
reads per pseudobulk sample	338173184.40	203410517.30	68383073.2	3521534.10	79433.100	17651.800
UMIs per pseudobulk sample	57232328.40	50090217.10	28800440.9	2054857.60	41964.600	4253.200
genes per pseudobulk sample	19504.57	19175.33	18257.5	14391.97	6514.767	1674.267

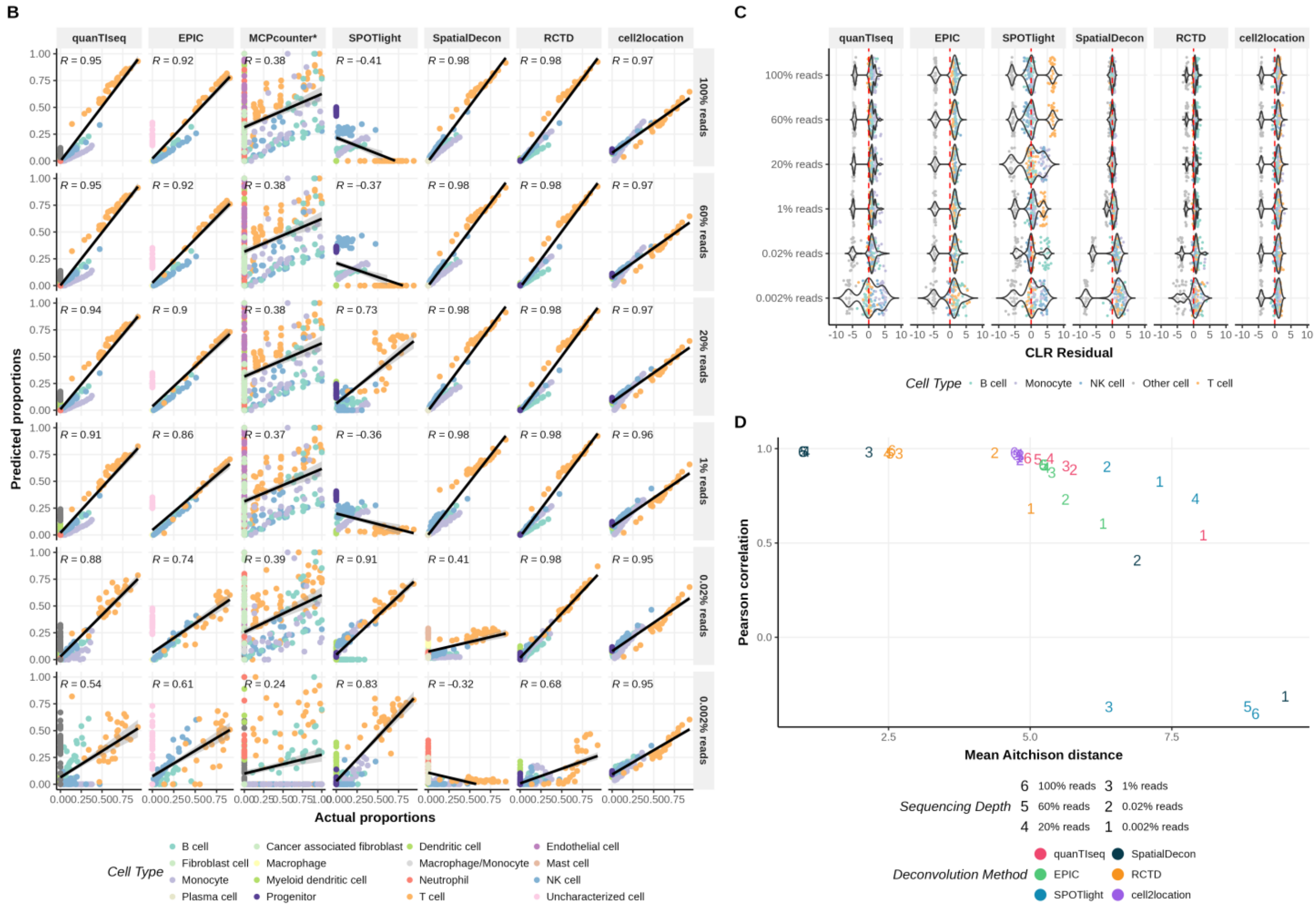

Fig. S2: Impact of sequencing depth the deconvolution performance in bone marrow pseudo-bulk samples.

(A) Reads were randomly sampled from the FASTQ files to obtain 100%, 60%, 20%, 1%, and 0.02% and 0.002% of the original total reads. CellRanger was run for each subsample, and cell annotations were transferred based on clustering from the 100% reads sample. These data were then processed following the method described in Fig. 1B to generate pseudo-bulk samples.

(B) Correlations between actual and predicted cell proportions in the PBMC pseudo-bulk samples. Points are coloured according cell types predicted by each method (Additional file 1: Table S2). Pseudo-bulk samples used as a basis for deconvolution only contained B cells, T cells, monocytes and NK cells. PCC is indicated in each plot.

(C) Distribution of the CLR residuals for deconvolution results across different methods and sequencing depths. Dot colours represent cell types.

(D) PCC versus mean AD comparing deconvolution performance across sequencing depths.

A

Normalisation	Formula
Raw	$= x_{ij}$
TPM normalised x'_{ij}	$= \frac{x_{ij}}{\sum_{i=1}^{m} x_{ij}} \cdot 10^6$
Q3 normalised x'_{ij}	$= \frac{x_{ij}}{Q3_j} \cdot \exp\left(\frac{1}{n}\sum_{k=1}^{n} \ln(Q3_k)\right)$
Quantile normalised x'_{ij}	$= preprocessCore::normalize.quantiles(X)$

PBMC

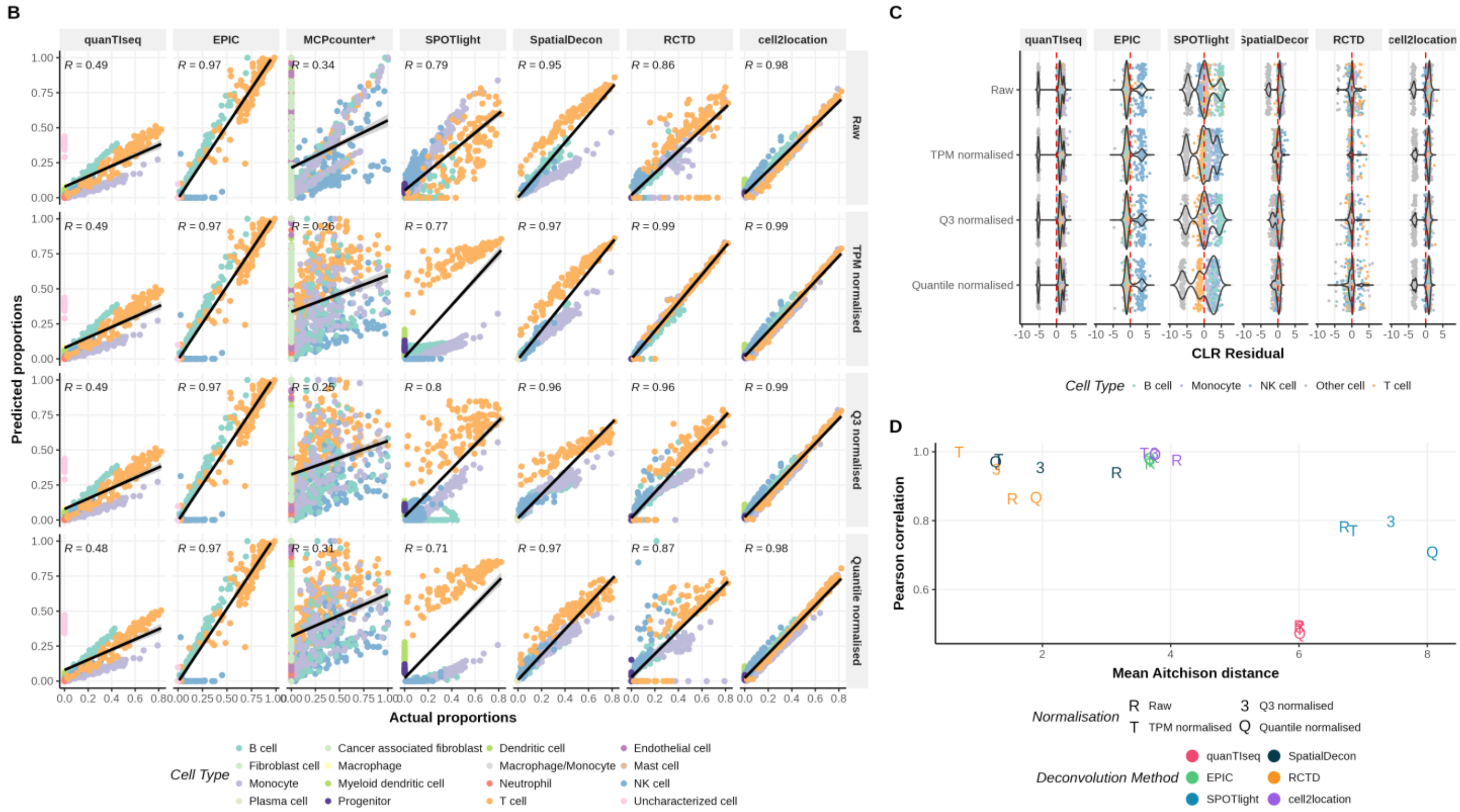

Healthy Bone Marrow

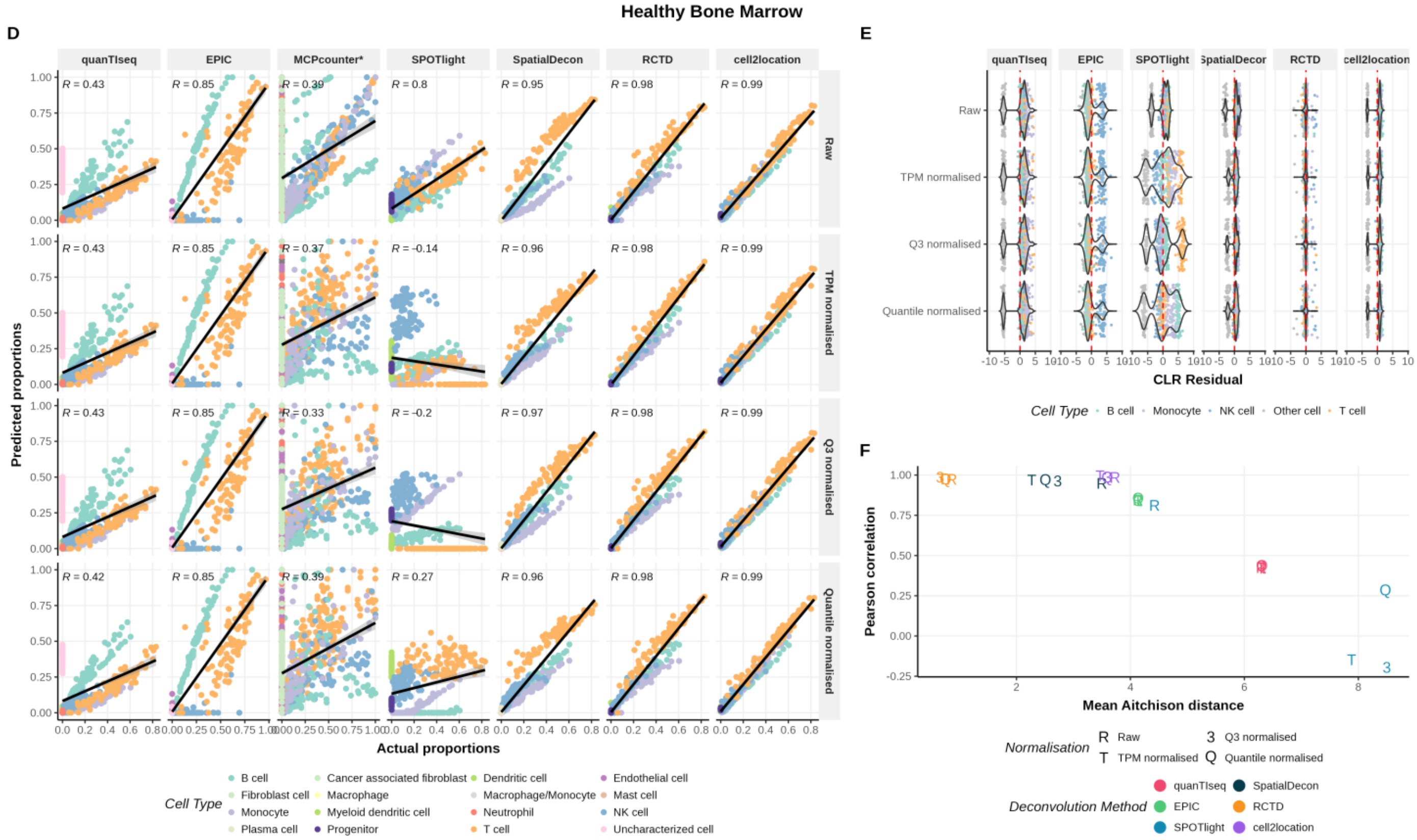

Fig. S3: Impact of normalisation strategies on predicted cell type proportions in PBMC and healthy bone marrow deconvolution

(A) Formulas used to normalize pseudo-bulk samples using four different normalisation strategies: raw (no normalisation), TPM normalised, Q3 normalisation, and quantile normalisation.

(B, E) Correlation between actual and predicted proportions of cells in the PBMC **(B)** and healthy bone marrow **(E)** pseudo-bulk samples. Each plot facet displays the PCC. Data were normalised using strategies mentioned above. Points are coloured according cell types predicted by each method (Additional file 1: Table S2). Pseudo-bulk samples used as a basis for deconvolution only contained B cells, T cells, monocytes and NK cells.

(C, F) Distribution of CLR residuals for the four cell types in PBMC **(C)** and healthy bone marrow **(F)** pseudo-bulk samples. The colour of the dots denotes the cell type.

(D, G) PCC versus mean AD comparing different combinations of normalisation methods and deconvolution tools in PBMC **(D)** and healthy bone marrow **(G)** pseudo-bulk samples.

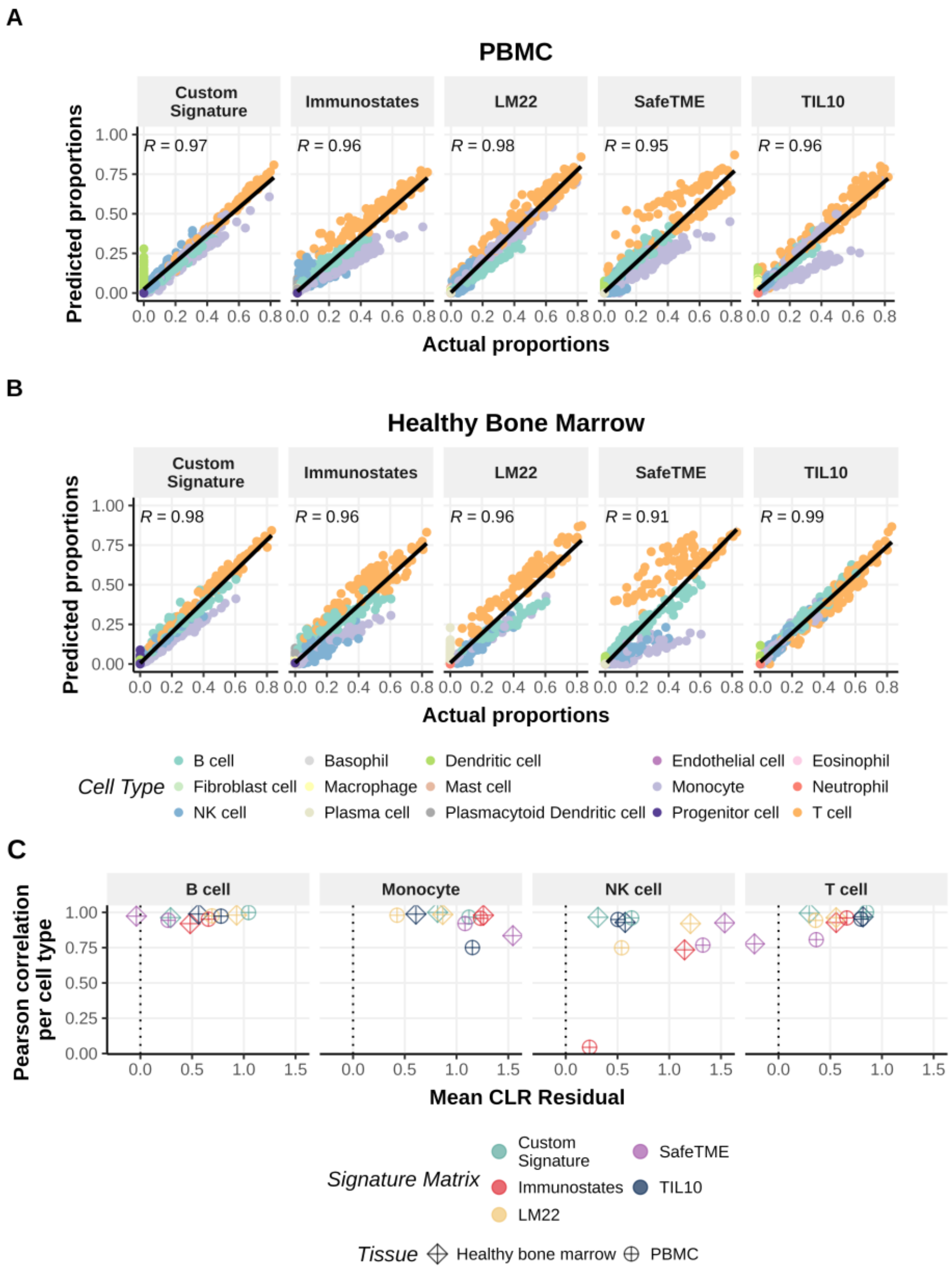

Fig. S4: Impact of choice of signature matrix on predicted proportions of cells of interest (using CIBERSORT)

(A, B) Correlations between actual and predicted proportions of cells in the (B) PBMC and (C) bone marrow pseudo-bulk samples. Points are coloured according cell types predicted by CIBERSORT. Pseudo-bulk samples used as a basis for deconvolution only contained B cells, T cells, monocytes and NK cells. **(C)** Performance evaluation of each signature matrix based on PCC versus mean CLR residuals across four cell types in PBMC and bone marrow pseudo-bulk samples.

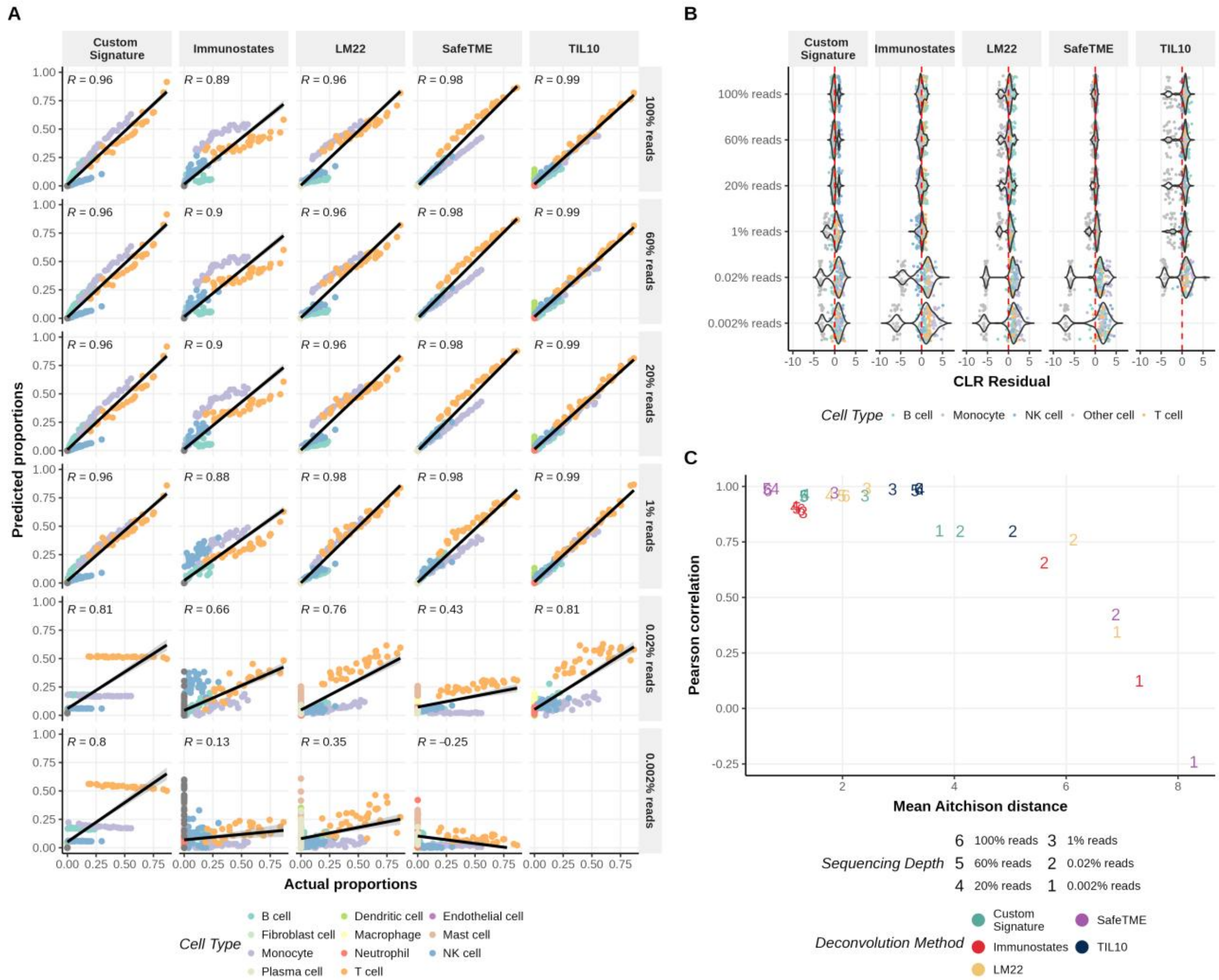

Fig. S5: Impact of choice of signature matrix on predicted proportions of cells of interest using SpatialDecon for samples of varying sequencing depths

(A) Correlations between actual and predicted cell proportions in the PBMC pseudo-bulk samples of varying sequencing depths when deconvoluted against five signature matrices using SpatialDecon. Points are coloured according cell types predicted by each method (Additional file 1: Table S2). Pseudo-bulk samples used as a basis for deconvolution only contained B cells, T cells, monocytes and NK cells. PCC is indicated in each plot. At 0.002% sequencing depth, TIL10 does not produce deconvolution results, as only 43 of the 170 genes in the TIL10 signature are present in the count matrix which was insufficient for reliable deconvolution by SpatialDecon. **(B)** Distribution of the CLR residuals for deconvolution results across different signature matrices and sequencing depths. Dot colours represent cell types. **(C)** PCC versus mean AD comparing deconvolution performance of signature matrices across sequencing depths.
